# The Thromboembolic Predictability of CHA_2_DS_2_-VASc Scores Using Different Echocardiographic Criteria for Congestive Heart Failure in Korean Patients with Nonvalvular Atrial Fibrillation

**DOI:** 10.3390/jcm11020300

**Published:** 2022-01-07

**Authors:** Albert Youngwoo Jang, Woong Chol Kang, Yae Min Park, Kyungeun Ha, Jeongduk Seo, Pyung Chun Oh, Kyounghoon Lee, Jeonggeun Moon

**Affiliations:** Division of Cardiology, Department of Internal Medicine, Gachon University Gil Medical Center, Incheon 21565, Korea; cardio_gil@gilhospital.com (A.Y.J.); ypruimin@gilhospital.com (Y.M.P.); back2heaven@naver.com (K.H.); jaidyseo@gilhospital.com (J.S.); likemed@gilhospital.com (P.C.O.); cardioman@gilhospital.com (K.L.)

**Keywords:** CHA2DS2-VASc score, diastolic function, E/E’, atrial fibrillation, congestive heart failure

## Abstract

The association between congestive heart failure (CHF) of the CHA_2_DS_2_-VASc scores and thromboembolic (TE) events in patients with atrial fibrillation (AF) is a topic of debate due to conflicting results. As the importance of diastolic impairment in the occurrence of TE events is increasingly recognized, it is crucial to evaluate the predictive power of CHA_2_DS_2_-VASc scores with C criterion integrating diastolic parameters. We analyzed 4200 Korean nonvalvular AF patients (71 years of age, 59% men) to compare multiple echocardiographic definitions of CHF. Various guideline-suggested echocardiographic parameters for systolic or diastolic impairment, including left ventricular ejection fraction (LVEF) ≤ 40%, the ratio of early diastolic mitral inflow velocity to early diastolic velocity of the mitral annulus (E/E’) ≥ 11, left atrial volume index > 34 mL/m^2^, and many others were tested for C criteria. Multivariate-adjusted Cox regression analysis showed that CHA_2_DS_2_-VASc score was an independent predictor for composite thromboembolic events only when CHF was defined as E/E’ ≥ 11 (hazard ratio, 1.26; *p* = 0.044) but not with other criteria including the original definition (hazard ratio, 1.10; *p* = 0.359). Our findings suggest that C criterion defined as diastolic impairment, such as E/E’ ≥ 11, may improve the predictive value of CHA_2_DS_2_-VASc scores.

## 1. Introduction

The presence of atrial fibrillation (AF) represents a major threat in cardiovascular (CV) health [1,2]. The powerful prognostic value of AF has shown to even nullify the predictive power of cardiac calcium, which is a more potent prognosticator compared with conventional CV risk factors [1]. The CHA_2_DS_2_-VASc score for risk stratification of thromboembolic (TE) events is the most widely used scoring system in patients with atrial AF worldwide [2]. The C criterion of the CHA_2_DS_2_-VASc scheme represents congestive heart failure (CHF) defined as evidence of heart failure (HF) or a left ventricular ejection fraction (LVEF) ≤ 40% [3]. However, the individual constituents of the definition, namely, the clinical diagnoses of CHF or low LVEF, have been questioned for its association with the risk of stroke [4].

The clinical diagnostic component of the definition, evidence of HF, may be unreliable due to difficulty differentiating HF from lung or pulmonary vascular diseases. As clinical diagnoses are open to interpretation, some studies define CHF based on International Classification of Diseases (ICD) codes [5] while others including textbooks [6] do not specify clear criteria, leading to the need for a less ambiguous definition for CHF in the CHA_2_DS_2_-VASc scheme. A Meta-analysis demonstrated that out of 12 studies that define CHF as clinical heart failure, only 3 showed an association between CHF and the occurrence of stroke in patients with AF [4].

Whether the echocardiographic component of CHF criteria, LVEF ≤ 40%, reflects the risk of stroke is also of significant importance. LVEF has been criticized for not appropriately reflecting cardiac contractility and being preload- or afterload-dependent [7]. The association between stroke and left ventricular systolic dysfunction, such as left ventricular fractional shortening ≤ 25%, LVEF ≤ 40%, or LVEF ≤ 50%, were evaluated in a meta-analysis of eight AF studies [3,8,9,10,11,12,13,14]. Although four studies showed a negative link, such studies had small sample sizes with small statistical power [4]. Recent data have also proposed that LVEF is weakly associated with stroke risk with or without anticoagulation [15,16]. Additionally, our understanding of CHF has evolved with the introduction of new concepts involving diastolic function such as HF with preserved ejection fraction (HFpEF) or diastolic dysfunction (DD) [17]. Left ventricular (LV) DD generally causes the LV filling pressure to increase, thereby dilating the left atrium (LA) [18]. The enlarged atrium and elevated pressure prompt blood stasis [19], which then promotes spontaneous echo contrasts (SEC) that predispose to thrombosis and stroke [20]. It was also recently proposed that diastolic attributes are incrementally impaired with elevated CHA_2_DS_2_-VASc scores in patients with nonvalvular AF (NVAF) [21]. Accordingly, echocardiographic indicators of impaired diastolic function are being increasingly recognized for its association with the incidence of stroke or elevated LV filling pressure in patients with AF.

The ambiguity of the original criteria and the emergence of diastolic impairment that is likely to be associated with stroke collectively prompt an investigation of novel echocardiographic criteria into the current C criterion. In this study, the performance of TE risk prediction of CHA_2_DS_2_-VASc schemes are evaluated by comparison of the traditional CHF definition (ICD code or LVEF ≤ 40%) with various guideline-suggested echocardiographic parameters of HFpEF or AF.

## 2. Methods

### 2.1. Study Sample

This study was approved by the institutional review board (IRB) of Gil Medical Center (GDIRB2018-305) and complied with the Declaration of Helsinki. Written informed consents were waived by the IRB due to the retrospective nature of study. We retrospectively analyzed the medical charts and our echocardiographic database of Korean AF patients at Gachon University Medical Center between 2011 and 2017. Inclusion criteria were: (1) Korean patients with AF during the transthoracic echocardiographic exam, during which AF was confirmed by A wave loss; (2) AF was confirmed by review of electrocardiogram between one-month prior to or after the echocardiography; and (3) age ≥ 18 years. Exclusion criteria were: (1) patients with more than moderate severity of either mitral or aortic valvular disease except for functional mitral regurgitation of any degree; and (2) history of surgery to the mitral or aortic valves. Comorbidities for evaluating CHA_2_DS_2_-VASc scores were obtained from the ICD codes of the medical records of each individual at the time of transthoracic echocardiography (TTE) exam, as described previously [21].

### 2.2. Echocardiography

We analyzed the two-dimensional transthoracic echocardiography data at initial presentation of AF. The LVEF, left ventricular end diastolic volume and left ventricular end systolic volume was calculated using the modified Simpson’s method. Left ventricular end-diastolic dimension (LVEDD) and left ventricular end-systolic dimension was measured in the two-dimensional mode of the parasternal long axis view. Maximal left atrial (LA) volume was calculated using the prolate ellipsoid model3. LA volume index (LAVI) was defined as LA volume indexed to the body surface area. In the apical window view, pulsed Doppler sample volume sized 1–2 mm was located at the tip of mitral valve, where mitral inflow velocities were measured. The peak early diastolic filling velocity (E) was divided by the early diastolic mitral annulus velocity (E’) measured by Doppler tissue imaging at the medial mitral annulus for E/E’ calculation. Detailed information regarding echocardiography were described previously [21].

### 2.3. Definitions of Various C Criteria

**Figure 1:**
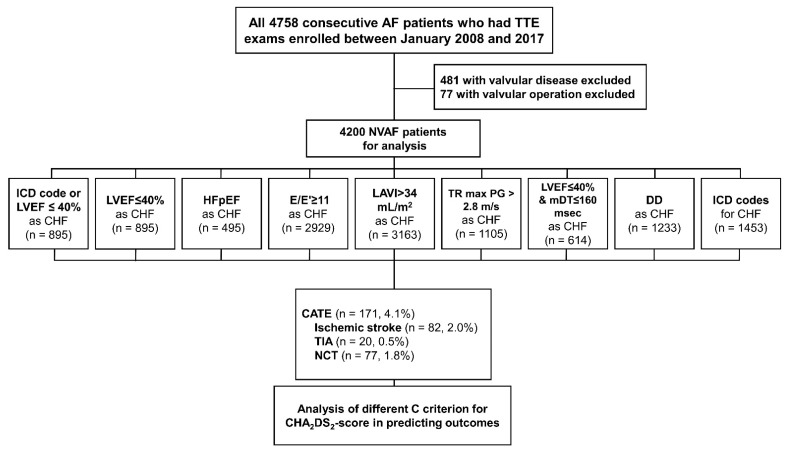
Diagram for enrollment: After the exclusion of 558 patients with valvular diseases, 4200 nonvalvular atrial fibrillation patients were analyzed. Multiple definitions including the original criteria were subjected were analyzed through multivariate Cox regression analysis for CATE. AF, atrial fibrillation; TTE, transthoracic echocardiography; NVAF, nonvalvular atrial fibrillation; NCT, non-cerebral thromboembolism. Other abbreviations are listed in Table 1.

The main comparator of this study was the original definition of CHF in the CHA_2_DS_2_-VASc score scheme, which was clinical HF or systolic dysfunction [3]. We defined clinical HF as patients with ICD codes for CHF and systolic dysfunction as LVEF ≤ 40% by echocardiography. Various guideline-suggested definitions of diastolic impairment either for AF or non-AF were also adopted and evaluated [17]. Tested echocardiographic criteria were, E/E’ ≥ 11 [22], mitral deceleration time (mDT) ≥ 160 ms in patients with reduced systolic function (LVEF ≤ 40% and mDT ≥ 160) [23], LVEF ≤ 40%, HFpEF, DD [17], or individual components of DD (TR max PG > 2.8 m/s or LAVI > 34 mL/m^2^). There are various guideline-suggested indicators for DD in patients with AF [17]. Sohn et al. suggested mitral annular E/E’ ≥ 11 as an indicator of elevated filling pressure, which is currently recommended in the American and European guideline for DD [17,22]. The mDT ≥ 160 in LVEF ≤ 40% was also recommended as a predictor for DD in AF patients [17,23]. In the American and European echocardiographic guideline for DD, DD is defined as more than two of the following four components: LAVI > 34 mL/m^2^, TR max PG ≥ 2.8 m/s, septal E’ velocity < 7 cm/s, and average E/E’ ≥ 15 in subjects with normal sinus rhythm [17]. We modified this definition by replacing average E/E’ ≥ 15 for mitral annular E/E’ ≥ 11, because the original definition of DD was for patients with normal sinus rhythm but not AF, and since most of our data regarding E/E’ were derived by mitral annular velocity and not average E/E’. HFpEF was defined as a combination of preserved systolic function (LVEF ≥ 50%) and the presence of DD. Clinically diagnosed CHF was defined as patients with relevant ICD codes for CHF. Patient selection and the sub-division process is presented in Figure 1.

### 2.4. Medication and Outcome Assessment

Concurrent medications taken during the follow-up period were assessed by chart review. The follow-up duration was cut to two years because there was a concern of data quality after two years since over 70% (2941 out of 4200) of patients were lost after two years. The duration of anticoagulant use was also evaluated. Anticoagulation was defined as the administration of a vitamin K antagonist or a novel oral anticoagulant. The primary endpoint was composite adverse thromboembolic events (CATE), which was defined as a composite of ischemic stroke, transient ischemic attack (TIA), and non-cerebral thromboembolism (NCT). Patients with more than one constituent endpoints were regarded as having one CATE; although, the final follow-up date was assigned to the endpoint that occurred earlier.

### 2.5. Statistical Analysis

The data analysis was performed using SPSS Statistics for Windows version 23.0 (IBM Corp., Armonk, NY, USA). Continuous normally distributed data are expressed as mean ± standard deviation, whereas continuous non-normally distributed data are given as a median and interquartile range (IQR). The continuous normally and non-normally distributed data between groups were compared by the two-sample *t*-test and Mann–Whitney U-test, respectively. One-way analysis of variance was applied to compare intergroup differences in normally distributed variables. Categorical variables were analyzed by Pearson’s χ^2^ test. To evaluate the independent effect of CHA_2_DS_2_-VASc scores with different definitions for CHF on outcomes, we performed a stepwise multivariate Cox proportional hazards regression model. We included parameters either clinically relevant or statistically significant in the univariate analysis for subsequent multivariate analysis. CHA_2_DS_2_-VASc scores using other CHF criteria were divided into three categories based on scores (0–1, 2–4, and 5 or above) and were defined as ordinal variables in the Cox model. The adjusted hazards ratio (HR) and 95% confidence interval (CI) of each CHA_2_DS_2_-VASc scores category were adjusted for left ventricular hypertrophy, cardioversion, or radiofrequency catheter ablation (RFCA), anticoagulation, anticoagulation duration, and the use of antiplatelet, angiotensin-converting enzyme/angiotensin receptor blocker antagonist (ACEi/ARB), beta-blockers (BB), calcium channel blockers, antiarrhythmics, and diuretics. Longitudinal data for outcomes were plotted using the Kaplan–Meier estimates with the log-rank test. We also performed multivariate linear regression to exclude variables with multicollinearity among adjusting variables for multivariate-Cox regression. C-statistics were also evaluation for each CHA_2_DS_2_-VASc score categories using different CHF definitions for predicting CATE.

## 3. Results

### 3.1. Baseline Characteristics

The study comprised 4200 NVAF patients with a mean of 71 years of age, among whom 2487 (59%) were men (Table 1). The mean CHA_2_DS_2_-VASc score of the total population was 3.2 ± 1.9. Different criteria including the original definition, LVEF ≤ 40%, HFpEF, E/E’ ≥ 11, LAVI > 34 mL/m^2^, TR max PG ≥ 2.8 m/s, DD, LVEF ≤ 40% and mDT ≤ 160 ms, and ICD codes were used to define CHF (Table 1 and Figure 1). Of the total patients, 34% were on anticoagulation for a mean duration of 4.4 ± 8.2 months, while 3% of patients were subjected to RFCA or cardioversion. Patient with CATE were older and more likely men than those without CATE (CATE(−)). The mean CHA_2_DS_2_-VASc score was comparable between the two groups. Additionally, patients with CATE (CATE(+)) group were significantly less likely to take anticoagulants, antiplatelets, antiarrhythmics, BB, ACEi/ARB, and diuretics than the CATE(−) group (Table 1). Demographic data among different CHA_2_DS_2_-VASc score groups are shown in Supplementary Table S1.

### 3.2. Clinical Outcomes 

Clinical outcomes of the total population are shown in Table 1. The median follow-up duration was 10.6 months (IQR, 2.0–24.0). CATE occurred in 171 patients (4.1%). A total of 82 (2.0%), 20 (0.5%), and 77 (1.8%) patients had ischemic stroke, TIA, and NCT, respectively.

### 3.3. Baseline Echocardiography Findings

The baseline echocardiography characteristics in the overall, CATE(+) and CATE(−) populations are shown in Table 2. The LVEF, LAVI, E/E’, TR max PG, and mDT of the total population were 52%, 49 mL/m^2^, 13.4, 2.5 m/s, and 119 ms, respectively. The CATE(+) group had significantly higher LV end diastolic diameter/body surface area and TR max PG than the CATE(−) group (Table 2). Echocardiographic features among CHA_2_DS_2_-VASc score groups are presented in Supplementary Table S1.

### 3.4. Multivariate Analysis Using Different Definitions for CHF

Covariates significant in the univariate Cox regression analysis (Supplementary Table S2) was included in the multivariate analysis. The multivariate-adjusted HR and 95% CI values of the different CHA_2_DS_2_-VASc score categories were assessed using different criteria for CHF (Table 3). When CHF was defined as the original definition, the CHA_2_DS_2_-VASc score category was not a significant predictor for CATE. However, when defined as E/E’ ≥ 11, CHA_2_DS_2_-VASc scores was an independent predictor. When other definitions with systolic or diastolic implications, such as LVEF ≤ 40%, HFpEF, LAVI > 34 mL/m^2^, TR max PG ≥ 2.8 m/s, LVEF ≤ 40% and mDT ≥ 160 ms, or DD were used, the CHA_2_DS_2_-VASc scores were not independent predictors for CATE. Table 4 shows the adjusted HR and 95% CI of all covariates included in each multivariate Cox regression model. The use of antiplatelets, ACEi/ARBs, and diuretics were robustly associated with favorable outcomes in all models.

Additionally, we did the stepwise multivariate analysis after excluding patients administered with anticoagulation because the short duration and low percentage of anticoagulation might be confounding (Supplementary Table S3). We analyzed a total of 2752 patients, among which 143 CATE occurred. Interestingly, the *p* value of the CHA_2_DS_2_-VASc scores categories was the lowest when CHF was defined as E/E’ ≥ 11 consistent with the analysis without patients with anticoagulation were not excluded (Supplementary Table S3).

### 3.5. Survival Analysis

**Figure 2:**
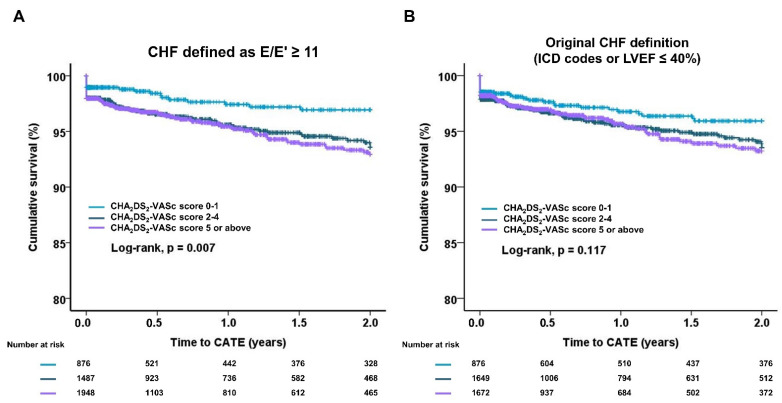
Kaplan–Meier survival analysis of different definitions for CHF: CHF of CHA_2_DS_2_-VASc scores was defined as E/E’ ≥ 11 (**A**) and as originally described (**B**). CHA_2_-DS_2_-VASc score categories show significantly increasing risk of CATE with increasing scores when CHF is defined as E/E’ ≥ 11 (log-rank test, *p* = 0.007), but not when defined by the original definition (log-rank, *p* = 0.117). LVEF, left ventricular ejection fraction.

Kaplan–Meier survival plots of E/E’ ≥ 11 or the original definition (ICD code or LVEF ≤ 40%) as CHF are shown in Figure 2A,B. CHA_2_DS_2_-VASc score categories showed a significantly increasing risk of CATE with increasing scores when CHF was defined as E/E’ ≥ 11, but not with the original definition (Figure 2A,B).

### 3.6. C-Statistics Analysis

The C-statistics of each CHA_2_DS_2_-VASc score categories are shown in Supplementary Table S4. Although the c-statistics were relatively low, the c-statistics of the CHA_2_DS_2_-VASc scores when CHF was as E/E′ ≥ 11 was the highest among other definitions.

### 3.7. Discussion/Conclusions

In this study, we found that CHA_2_DS_2_-VASc score was an independent predictor for CATE when CHF was E/E’ ≥ 11 but not when defined as originally (ICD code or LVEF ≤ 40%) in the Korean NVAF population. When other guideline-suggested parameters for HFpEF/HFrEF or AF were tested for the C criterion, such as LVEF ≤ 40%, HFpEF, DD, LAVI > 34 mL/m^2^, TR max PG ≥ 2.8 m/s, LVEF ≤ 40% and mDT ≥ 160 ms, or ICD codes, CHA_2_DS_2_-VASc scores were not predictors for CATE.

The concept of LVEF was originally proposed by a seminal study in the 1960s to reliably measure contractility [24]. LVEF is now widely used as a key criterion for classifying HF with or without impaired contractility (HFrEF or HFpEF, respectively). Recently, however, LVEF has been criticized for not truly reflecting contractility [7]. The argument centers on the LVEF being load-dependent, as rise in preload and afterload may increase the stroke volume (SV) and contractile function, while LVEF may be unchanged based on the Frank–Starling mechanism. Alternatively, the hypertrophic and dilated heart with the same reduced SV may have very different LVEF values [7]. This suggests that LVEF can be misleading. Additionally, the association between impaired systolic function, typically LVEF ≤ 40%, and stroke has also been challenged. A recent study proposed that the presence of impaired systolic function in NVAF patients was not associated with the risk of thromboembolic events in patients with [15] or without oral anticoagulation [16]. These examples collectively propose that LVEF may not be a reliable marker of the pathophysiology of HF or stroke.

Although risk scoring systems were originally devised to conveniently use clinical information, there is a growing need for clearly defined echocardiography criteria for CHF. The original definition of CHF in the CHA_2_DS_2_-VASc schema, leaves clinical HF (ICD codes) open to being construed arbitrarily. These problems are highlighted by only 29.8% (433 patients) and 36.7% (533 patients) of patients with a clinical diagnosis (ICD code) of CHF (1454 patients) having HFrEF or HFpEF, respectively, in our study. These findings propose the unreliable nature of clinical diagnoses or LVEF to predict TE events in AF patients and question the adequacy of including such criteria in the definition of CHA_2_DS_2_-VASc scores.

Diastolic echocardiographic parameters such as E/E’ ≥ 11 may be considered a definition of CHF for the CHA_2_DS_2_-VASc scores. Impaired diastolic function is associated with elevated LV filling pressure, stroke, and high CHA_2_DS_2_-VASc scores [18]. Among the multiple diastolic parameters that we tested here, E/E’ showed the most promising findings. Seminal studies have revealed that the diastolic parameter E/E’ has a good correlation with LV filling pressure with great reliability [22]. E/E’ has been demonstrated as an independent predictor of LAA mechanical function and stroke in AF patients [25,26]. It was also proposed that E/E’ is incrementally impaired with the increase in CHA_2_DS_2_-VASc scores of NVAF patients [21]. E/E’ is also thoroughly proven to have good clinical validity and reliability in AF patients [23,27,28]. Accordingly, screening the LV filling pressure through an echocardiographic evaluation of E/E’ in NVAF patients may be crucial for improving the performances of risk-stratifying schemas.

Several aspects may have contributed to the comparable LA dimensions between the CATE(+) and CATE(−) group. First, the presence of AF in all patients may have confounding effects. Four factors that are generally accepted as determinants of LAVI are the presence of AF, mitral regurgitation, DD, and LV remodeling [29]. Since patients in this cohort all had AF, the LA enlargement may have been mainly driven by the presence of AF more so than other factors. The strong effect of AF may disrupt the conventionally observed phenomenon, more stroke in larger LA. Second, the heterogeneity of our cohort may contribute to such counter-intuitive results. The present cohort comprises those with primary/secondary stroke prevention and subjects visiting for peri-operative risk evaluation or health screenings. The admixture of underlying pathophysiologies may confound the results of LA dimensions. Third, the difficulties in echocardiographic imaging in the presence of CHF could influence the results. Of the total patients, 35% had a history of CHF based on ICD codes. As pointed out by a study by Faganello and colleagues, integrative modality imaging is required for a more precise assessment of heart failure [30]. The intrinsic limitations of echocardiographic imaging alone may possibly explain the similar LA dimension between the CATE+ and CATE− groups.

The current study has several limitations. The difference in predictive value of the CHA_2_DS_2_-VASc scores among different populations has not been validated, bringing into question the generalizability of our findings to all populations. For example, in a study predominantly enrolled with Caucasians, LVEF or E/E’ was not predictive of TE events; although, E/E’ > 13 was significantly predictive of death [31]. Since the current study is a single-center observational study, further large-scale multi-national cohorts using echocardiographic criteria are necessary for the establishment of our findings. A relatively low percentage (34%) of patients were on anticoagulants, and a short follow-up period may also be a limitation of the study. Since many of the patients undergoing the echocardiographic exam were those who visited a tertiary center for peri-operative evaluation or health screening, such patients were probably lost during follow up and were biased. This may have resulted in the incomplete information of the medication as well. Although these substantial drawbacks may be intrinsic limitations of a single-center observational study, we believe that our large sample size may compensate for such confounding factors. Furthermore, the patient population included in our study may be substantially biased, as they were mainly checked by echocardiography for either heart disease or peri-operative evaluation.

In this study: we found that CHA_2_DS_2_-VASc scores had the most promising performance for predicting CATE when E/E’ ≥ 11 was the definition for CHF in Korean NVAF patients. When other definitions, including the original definition, were used for CHF, the CHA_2_DS_2_-VASc scores were not predictive of CATE. These results lead to a demand for integrating a diastolic parameter into the current C criterion of the CHA_2_DS_2_-VASc scores.

## Figures and Tables

**Table 1:** Baseline characteristics and outcomes of total cohort.

	All (n = 4200)	CATE(−) (n = 4029)	CATE(+) (n = 171)	*p*
Demographic data				
Age (years)	71 ± 12	71 ± 12	73 ± 11	0.005
Men, n (%)	2487 (59)	2391 (59)	95 (56)	0.323
Congestive heart failure				
ICD code or LVEF ≤ 40%, n (%)	1962 (47)	1885 (47)	77 (45)	0.652
LVEF ≤ 40%, n (%)	895 (21)	858 (21)	37 (22)	0.924
HFpEF, n (%)	495 (12)	476 (12)	19 (11)	0.904
E/E’ ≥ 11, n (%)	2929 (71)	3800 (71)	129 (76)	0.168
LAVI>34 mL/m^2^, n (%)	3163 (77)	3029 (77)	134 (80)	0.197
TR max PG>2.8 m/s, n (%)	1105 (26)	1051 (26)	54 (32)	0.070
DD, n (%)	1233 (29)	1183 (29)	50 (29)	1.000
LVEF ≤ 40% and mDT ≤ 160 ms, n (%)	614 (15)	593 (15)	21 (13)	0.573
ICD codes for CHF, n (%)	1453 (35)	1400 (35)	53 (31)	0.326
Hypertension, n (%)	1641 (39)	1574 (39)	67 (39)	1.000
Diabetes mellitus, n (%)	751 (18)	723 (18)	28 (16)	0.684
Secondary prevention for TE, n(%)	908 (22)	876 (22)	32 (19)	0.200
Ischemic stroke, n (%)	792 (19)	769 (19)	23 (14)	0.072
TIA, n (%)	96 (2)	92 (2)	4 (2)	0.797
Systemic/pulmonary TE, n (%)	110 (3)	102 (3)	8 (5)	0.088
Peripheral arterial disease, n (%)	78 (2)	75 (2)	2 (1)	0.497
Myocardial infarction, n (%)	199 (5)	192 (5)	7 (4)	0.854
Aortic plaque, n (%)	110 (3)	102 (3)	8 (5)	0.088
Systolic blood pressure (mm Hg)	125 ± 27	125 ± 27	123 ± 24	0.513
Diastolic blood pressure (mm Hg)	74 ± 14	74 ± 14	72 ± 15	0.176
Heart rate (bpm)	89 ± 26	89 ± 26	86 ± 21	0.303
Height (cm)	162 ± 11	162 ± 11	160 ± 12	0.019
Weight (kg)	64 ± 11	64 ± 13	61 ± 16	0.003
BMI	24.4 ± 9.5	24.4 ± 7.6	25.3 ± 29.4	0.195
Mean CHA_2_DS_2_-VASc score	3.2 ± 1.9	3.2 ± 1.9	3.4 ± 1.7	0.227
Medication or Procedures				
RFCA/CV	120 (3)	119 (3)	1 (1)	0.094
Anticoagulation, n (%)	1449 (34)	1421 (35)	29 (16)	<0.001
VKA, n (%)	845 (20)	834 (21)	11 (6)	
NOAC, n (%)	604 (14)	587 (15)	17 (10)	
Duration (months)	4.4 ± 8.2	4.5 ± 8.3	2.1 ± 6.2	<0.001
Antiplatelet, n (%)	766 (18)	188 (21)	578 (17)	0.008
Duration (months)	2.1 ± 6.2	2.2 ± 6.3	0.8 ± 4.2	<0.001
Antiarrhythmics, n (%)	400 (10)	398 (10)	2 (1)	<0.001
Beta blockers, n (%)	1129 (27)	1104 (27)	25 (15)	<0.001
Calcium channel blockers, n (%)	289 (7)	283 (7)	6 (4)	0.088
ACEi/ARBs, n (%)	1385 (33)	1360 (34)	25 (15)	<0.001
Diuretics, n (%)	1288 (31)	1260 (31)	28 (16)	<0.001
2-year clinical events				
Median follow up (months)	10.6 (2.0–24.0)	11.3 (2.3–24.0)	1.5 (0–8.9)	<0.001
CATE, n (%)	171 (4.1)	0 (0)	171 (4.1)	<0.001
Ischemic stroke, n (%)	82 (2.0)	0 (0)	82 (48)	<0.001
TIA, n (%)	20 (0.5)	0 (0)	20 (11.7)	<0.001
NCT, n (%)	77 (1.8)	0 (0)	77 (45.0)	<0.001

CATE, composite adverse thromboembolic events; ICD, the International Statistical Classification of Diseases and Related Health Problem; LVEF ≤ 40%, left ventricular ejection fraction less than 40%; HFpEF, heart failure with preserved ejection fraction; DD, diastolic dysfunction; mDT, mitral deceleration time; E/E’, the ratio of early diastolic mitral inflow velocity to early diastolic velocity of the mitral annulus; LAVI, left atrial volume index; TR max PG, tricuspid regurgitation max pressure gradient; mDT, mitral deceleration times; CHF, congestive heart failure; TIA, transient ischemic attack; BMI, body mass index; RFCA, radiofrequency catheter ablation; CV, cardioversion; VKA, vitamin K antagonist; NOAC, new oral anticoagulant; ACEi, angiotensin converting enzyme inhibitor; ARB, angiotensin receptor blocker; NCT, non-cerebral thromboembolism.

**Table 2:** Baseline echocardiography characteristics in patients with or without primary endpoints.

	All (*n* = 4200)	CATE(−) (n = 4029)	CATE(+) (n = 171)	*p*
LVEF (%)	52 ± 16	52 ± 16	53 ± 15	0.952
LVEDD (mm)	50 ± 6	50 ± 6	50 ± 7	0.532
LVESD (mm)	35 ± 10	35 ± 10	35 ± 8	0.642
LVEDV (mL)	77 ± 37	77 ± 37	72 ± 35	0.182
LVESV (mL)	42 ± 31	43 ± 31	41 ± 30	0.523
BSA (m^2^)	1.7 ± 0.2	1.7 ± 0.2	1.6 ± 0.3	<0.001
LVEDD/BSA (mm/m^2^)	30 ± 5	30 ± 5	31 ± 5	0.012
LVESD/BSA (mm/m^2^)	21 ± 6	21 ± 6	22 ± 5	0.194
LVEDV/BSA (mL/m^2^)	46 ± 21	46 ± 21	44 ± 19	0.552
LVESV/BSA (mL/m^2^)	25 ± 18	25 ± 18	25 ± 17	0.848
LA diameter (mm)	45 ± 16	45 ± 16	45 ± 16	0.857
LVMI (g/m^2^)	97 ± 31	97 ± 31	100 ± 29	0.251
RWT	0.37 ± 0.12	0.37 ± 0.12	0.37 ± 0.08	0.716
LAVI (mL/m^2^)	49 ± 22	49 ± 23	49 ± 20	0.985
E/E’	13.4 ± 6.2	13.3 ± 6.1	13.8 ± 6.7	0.287
Septal E’ velocity (cm/s)	7 ± 3	7 ± 3	7 ± 2	0.217
TR velocity max (m/s)	2.5 ± 0.4	2.5 ± 0.4	2.6 ± 0.4	0.004
mDT (ms)	119 ± 114	119 ± 116	115 ± 70	0.728
LVH, n (%)	1370 (33)	1308 (33)	62 (36)	0.318

LVEF, ejection fraction; LVEDD, left ventricular end diastolic dimension; LVESD, left ventricular end systolic dimension; LVEDV, left ventricular end diastolic volume; LVESV, left ventricular end systolic volume; BSA, body surface area; LA, left atrial; LVMI, left ventricular mass index; LAVI, left atrial volume index; E/E’, the ratio of early diastolic mitral inflow velocity to early diastolic velocity of the mitral annulus; RWT, relative wall thickness; mDT, mitral deceleration time; LVH, echocardiographic left ventricular hypertrophy.

**Table 3:** Multivariable-adjusted Cox regression analysis of each CHA_2_DS_2_-VASc scores using different definitions for congestive heart failure in predicting composite thromboembolic events.

Definition of C Criterion of CHA2DS2-VASc Score	CATE		
	Adjusted HR	95% CI	*p*
ICD code or LVEF ≤ 40%	1.10	0.90–1.36	0.359
LVEF ≤ 40%	1.19	0.97–1.47	0.100
HFpEF	1.07	0.88–1.31	0.493
E/E’ ≥ 11	1.26	1.01–1.57	0.044 *
LAVI > 34 mL/m^2^	1.78	0.94–1.48	0.156
TR max PG > 2.8 m/s	1.14	0.93–1.40	0.211
LVEF ≤ 40% and mDT ≥ 160 ms	1.16	0.85–1.57	0.348
DD	1.14	0.93–1.40	0.204
ICD code	1.01	0.82–1.24	0.939

Adjusted for cardioversion or RFCA, anticoagulation, anticoagulation duration (per months), antiplatelet, ACEi/ARBs, beta blocker, calcium channel blocker, antiarrhythmics, and diuretics. All abbreviations are listed in Table 1. Adjusted hazard ratio and 95% confidence intervals of other adjusted variables are shown in Supplementary Table S2. * *p* < 0.05.

**Table 4:** All covariates in the multivariable Cox regression models in predicting composite adverse thromboembolic events.

	Model 1		Model 2		Model 3	
	Adjusted HR (95% CI)	*p*	Adjusted HR (95% CI)	*p*	Adjusted HR (95% CI)	*p*
CHA_2_DS_2_-VASc (E/E’ ≥ 11)	1.26 (1.01–1.57)	0.044 *	-	-	-	-
CHA_2_DS_2_-VASc (ICD code or LVEF ≤ 40%)	-	-	1.10 (0.90–1.36)	0.359	-	-
CHA_2_DS_2_-VASc (LVEF ≤ 40%)	-	-	-	-	1.19 (0.97–1.47)	0.100
RFCA/CV	0.86 (0.11–6.37)	0.856	0.81 (0.11–6.25)	0.840	0.80 (0.10–6.19)	0.833
Anticoagulation	0.54 (0.28–1.04)	0.064	0.55 (0.29–1.06)	0.074	0.54 (0.28–10.5)	0.068
Antiplatelets	0.34 (0.18–0.62)	0.001 *	0.33 (0.18–0.62)	<0.001 *	0.33 (0.18–0.62)	<0.001 *
Antiarrhythmics	0.17 (0.04–0.71)	0.016 *	0.16 (0.04–0.68)	0.157	0.16 (0.04–0.70)	0.015 *
ACEi/ARBs	0.41 (0.26–0.63)	<0.001 *	0.42 (0.27–0.64)	<0.001 *	0.41 (0.27–0.64)	<0.001 *
BBs	0.77 (0.50–1.20)	0.250	0.77 (0.49–1.20)	0.241	0.77 (0.50–1.20)	0.246
CCBs	0.51 (0.22–1.15)	0.204	0.52 (0.23–1.17)	0.115	0.52 (0.23–1.17)	0.115
Diuretics	0.56 (0.36–0.85)	0.006 *	0.56 (0.37–0.85)	0.006 *	0.55 (0.36–0.84)	0.006 *
Anticoagulation duration (months)	0.98 (0.95–1.02)	0.391	0.98 (0.94–1.02)	0.359	0.98 (0.95–1.02)	0.381

E/E’, the ratio of early diastolic mitral inflow velocity to early diastolic velocity of the mitral annulus; ICD, the International Statistical Classification of Diseases and Related Health Problem; LVEF, left ventricular ejection fraction; RFCA: radiofrequency catheter ablation; CV, cardioversion; ACEi/ARB, angiotensin converting enzyme inhibitor/angiotensin receptor blocker; BB: beta blocker, CCB, calcium channel blocker. * *p* < 0.05.

## Data Availability

Data are not available due to strict patient health information regulations.

## Supplementary Materials

The following supporting information can be downloaded at: https://www.mdpi.com/article/10.3390/jcm11020300/s1, Table S1: Baseline demographic and echocardiographic features of CHA2DS2-VASc score categories; Table S2: Univariate Cox regression models for CATE; Table S3: Multivariable-adjusted Cox regression analysis of each CHA2DS2-VASc scores using different definitions for congestive heart failure in predicting composite thromboembolic events after excluding patients administered with anticoagulation; Table S4: C-statistics of different definitions used for congestive heart failure of the CHA2DS2-VASc scores in predicting composite adverse thromboembolic events.

## References

[1] Mazzone C., Cioffi G., Di Nora C., Barbati G., Guidetti F., Faggiano P., Gaibazzi N., Faganello G., Borca E.C., Di Lenarda A. (2018). Prognostic role of cardiac calcifications in primary prevention: A powerful marker of adverse outcome highly dependent on underlying cardiac rhythm. Int. J. Cardiol..

[2] Ikeda T. (2014). Which Score Should Be Used for Risk Stratification of Ischemic Stroke in Patients With Atrial Fibrillation. Circ. J..

[3] Lip G.Y., Nieuwlaat R., Pisters R., Lane D.A., Crijns H.J. (2010). Refining clinical risk stratification for predicting stroke and thromboembolism in atrial fibrillation using a novel risk factor-based approach: The euro heart survey on atrial fibrillation. Chest.

[4] Pisters R., Lane D.A., Marin F., Camm A.J., Lip G.Y.H. (2012). Stroke and Thromboembolism in Atrial Fibrillation. Circ. J..

[5] Friberg L., Lund L.H. (2018). Heart failure: A weak link in CHA2 DS2 -VASc. ESC Heart Fail.

[6] Jameson J.L. (2018). Harrison’s Principles of Internal Medicine.

[7] Konstam M.A., Abboud F.M. (2017). Ejection Fraction: Misunderstood and Overrated (Changing the Paradigm in Categorizing Heart Failure). Circulation.

[8] (1992). Predictors of thromboembolism in atrial fibrillation: I. Clinical features of patients at risk. The Stroke Prevention in Atrial Fibrillation Investigators. Ann. Intern. Med..

[9] Stroke Prevention in Atrial Fibrillation I. (1995). Risk factors for thromboembolism during aspirin therapy in patients with atrial fibrillation: The stroke prevention in atrial fibrillation study. J. Stroke Cerebrovasc. Dis..

[10] Aronow W.S., Ahn C., Kronzon I., Gutstein H. (1998). Risk factors for new thromboembolic stroke in patients > or = 62 years of age with chronic atrial fibrillation. Am. J. Cardiol..

[11] Stollberger C., Chnupa P., Kronik G., Brainin M., Finsterer J., Schneider B., Slany J. (1998). Transesophageal echocardiography to assess embolic risk in patients with atrial fibrillation. Ann. Intern. Med..

[12] Hart R.G., Pearce L.A., McBride R., Rothbart R.M., Asinger R.W. (1999). Factors associated with ischemic stroke during aspirin therapy in atrial fibrillation: Analysis of 2012 participants in the SPAF I-III clinical trials. The Stroke Prevention in Atrial Fibrillation (SPAF) Investigators. Stroke.

[13] Hart R.G., Pearce L.A., Rothbart R.M., McAnulty J.H., Asinger R.W., Halperin J.L. (2000). Stroke with intermittent atrial fibrillation: Incidence and predictors during aspirin therapy. Stroke Prevention in Atrial Fibrillation Investigators. J. Am. Coll. Cardiol..

[14] Stollberger C., Chnupa P., Abzieher C., Langer T., Finsterer J., Klem I., Hartl E., Wehinger C., Schneider B. (2004). Mortality and rate of stroke or embolism in atrial fibrillation during long-term follow-up in the embolism in left atrial thrombi (ELAT) study. Clin. Cardiol..

[15] McMurray J.J., Ezekowitz J.A., Lewis B.S., Gersh B.J., van Diepen S., Amerena J., Bartunek J., Commerford P., Oh B.H., Harjola V.P. (2013). Left ventricular systolic dysfunction, heart failure, and the risk of stroke and systemic embolism in patients with atrial fibrillation: Insights from the ARISTOTLE trial. Circ. Heart Fail..

[16] Sandhu R.K., Hohnloser S.H., Pfeffer M.A., Yuan F., Hart R.G., Yusuf S., Connolly S.J., McAlister F.A., Healey J.S. (2015). Relationship between degree of left ventricular dysfunction, symptom status, and risk of embolic events in patients with atrial fibrillation and heart failure. Stroke.

[17] Nagueh S.F., Smiseth O.A., Appleton C.P., Byrd B.F., Dokainish H., Edvardsen T., Flachskampf F.A., Gillebert T.C., Klein A.L., Lancellotti P. (2016). Recommendations for the Evaluation of Left Ventricular Diastolic Function by Echocardiography: An Update from the American Society of Echocardiography and the European Association of Cardiovascular Imaging. J. Am. Soc. Echocardiogr..

[18] Tsang T.S., Gersh B.J., Appleton C.P., Tajik A.J., Barnes M.E., Bailey K.R., Oh J.K., Leibson C., Montgomery S.C., Seward J.B. (2002). Left ventricular diastolic dysfunction as a predictor of the first diagnosed nonvalvular atrial fibrillation in 840 elderly men and women. J. Am. Coll. Cardiol..

[19] Ha J.W., Lee B.K., Kim H.J., Pyun W.B., Byun K.H., Rim S.J., Chung N. (2001). Assessment of left atrial appendage filling pattern by using intravenous administration of microbubbles: Comparison between mitral stenosis and mitral regurgitation. J. Am. Soc. Echocardiogr..

[20] Goldman M.E., Pearce L.A., Hart R.G., Zabalgoitia M., Asinger R.W., Safford R., Halperin J.L. (1999). Pathophysiologic correlates of thromboembolism in nonvalvular atrial fibrillation: I. Reduced flow velocity in the left atrial appendage (The Stroke Prevention in Atrial Fibrillation [SPAF-III] study). J. Am. Soc. Echocardiogr..

[21] Jang A.Y., Yu J., Park Y.M., Shin M.S., Chung W.J., Moon J. (2018). Cardiac Structural or Functional Changes Associated with CHA2DS2-VASc Scores in Nonvalvular Atrial Fibrillation: A Cross-Sectional Study Using Echocardiography. J. Cardiovasc. Imaging.

[22] Sohn D.W., Song J.M., Zo J.H., Chai I.H., Kim H.S., Chun H.G., Kim H.C. (1999). Mitral annulus velocity in the evaluation of left ventricular diastolic function in atrial fibrillation. J. Am. Soc. Echocardiogr..

[23] Nagueh S.F., Kopelen H.A., Quinones M.A. (1996). Assessment of left ventricular filling pressures by Doppler in the presence of atrial fibrillation. Circulation.

[24] Holt J.P., Rhode E.A., Peoples S.A., Kines H. (1962). Left ventricular function in mammals of greatly different size. Circ. Res..

[25] Kim T.H., Shim C.Y., Park J.H., Nam C.M., Uhm J.S., Joung B., Lee M.H., Pak H.N. (2016). Left ventricular diastolic dysfunction is associated with atrial remodeling and risk or presence of stroke in patients with paroxysmal atrial fibrillation. J. Cardiol..

[26] Lee J.S., Shim C.Y., Wi J., Joung B., Ha J.W., Lee M.H., Pak H.N. (2013). Left ventricular diastolic function is closely associated with mechanical function of the left atrium in patients with paroxysmal atrial fibrillation. Circ. J..

[27] Kotecha D., Mohamed M., Shantsila E., Popescu B.A., Steeds R.P. (2017). Is echocardiography valid and reproducible in patients with atrial fibrillation? A systematic review. Europace.

[28] Matsukida K., Kisanuki A., Toyonaga K., Murayama T., Nakashima H., Kumanohoso T., Yoshifuku S., Saigo M., Abe S., Hamasaki S. (2001). Comparison of transthoracic Doppler echocardiography and natriuretic peptides in predicting mean pulmonary capillary wedge pressure in patients with chronic atrial fibrillation. J. Am. Soc. Echocardiogr..

[29] Rossi A., Cicoira M., Zanolla L., Sandrini R., Golia G., Zardini P., Enriquez-Sarano M. (2002). Determinants and prognostic value of left atrial volume in patients with dilated cardiomyopathy. J. Am. Coll. Cardiol..

[30] Faganello G., Doimo S. (2017). Cardiac imaging in patients with acute or chronic heart failure. Minerva Cardioangiol..

[31] Gupta D.K., Giugliano R.P., Ruff C.T., Claggett B., Murphy S., Antman E., Mercuri M.F., Braunwald E., Solomon S.D. (2016). The Prognostic Significance of Cardiac Structure and Function in Atrial Fibrillation: The ENGAGE AF-TIMI 48 Echocardiographic Substudy. J. Am. Soc. Echocardiogr..

