# Supplementary material for: The Thromboembolic Predictability of CHA2DS2-VASc Scores Using Different Echocardiographic Criteria for Congestive Heart Failure in Korean Patients with Nonvalvular Atrial Fibrillation
Source: J Clin Med. 2022 Jan 7;11(2):300. doi: 10.3390/jcm11020300 (PMC8781364; doi:10.3390/jcm11020300)
Supplement: Supplementary file 1 [file jcm-11-00300-s001.zip › jcm-1534590-supplementary.pdf]

SUPPLEMENTAL MATERIAL

TITLE: The thromboembolic predictability of CHA_2DS_2-VASc scores using different echocardiographic criteria for congestive heart failure in Korean patients with nonvalvular atrial fibrillation

Albert Youngwoo Jang, MD, MS[1]; Woong Chol Kang, MD, PhD[1*]; Yae Min Park, MD, PhD[1]; Jeongduk Seo, MD, MS[1]; Kyungeun Ha , MD, MS[1]; Pyung Chun Oh, MD, PhD[1]; Kyounghoon Lee, MD, PhD[1]; Jeonggeun Moon, MD, PhD[1*]
[1]Division of Cardiology, Department of Internal Medicine, Gachon University Gil Medical Center, Incheon, Republic of Korea

Correspondence:
Woong Chol Kang, MD, PhD
Professor
Cardiology Division, Department of Internal Medicine, Gachon University Gil Medical Center
Address: 21 Namdong-daero 774beon-gil, Namdong-gu, Incheon 21565, Republic of Korea
Tel: +82-32-460-8305 (Office); Fax: +82-32-469-1906; E-mail: kangwch@gilhospital.com

Jeonggeun Moon, MD, PhD
Associate Professor
Cardiology Division, Department of Internal Medicine, Gachon University Gil Medical Center
Address: 21 Namdong-daero 774beon-gil, Namdong-gu, Incheon 21565, Republic of Korea
Tel: +82-32-460-3054; Fax: +82-32-469-1906; E-mail: jeff76@gilhospital.com

Number of supplementary data: 4 tables

Supplementary Table S1. Baseline demographic and echocardiographic features of CHA_2DS_2-VASc score categories.

	All (n = 4,200)	CHA_2DS_2-VASc Score 0-1 (n=877)	CHA_2DS_2-VASc Score 2-3 (n=1,650)	CHA_2DS_2-VASc Score 4-9 (n=1,673)	P
Demographic data					
**Age (years)**	71 ± 12	59 ± 9	72 ± 10	78 ± 7	<0.001*
**Men, n (%)**	2,494 (60)	932 (91)	1,081 (61)	481 (34)	<0.001*
**CHF (ICD code or LVEF ≤ 40%)**	1963 (47)	168 (19)	703 (43)	1092 (65)	<0.001*
**Hypertension, n (%)**	1,646 (39)	181 (18)	617 (35)	848 (61)	<0.001*
**Diabetes mellitus, n (%)**	751 (18)	54 (5)	228 (13)	469 (34)	<0.001*
**Stroke, n (%)**	792 (19)	0 (0)	129 (7)	663 (47)	<0.001*
**TIA, n (%)**	96 (2)	0 (0)	21 (1)	75 (5)	<0.001*
**Thromboembolism, n (%)**	110 (3)	0 (0)	3 (0)	107 (8)	<0.001*
**Peripheral arterial disease, n (%)**	78 (2)	6 (1)	18 (1)	54 (4)	<0.001*
**Myocardial infarction, n (%)**	199 (5)	12 (1)	48 (3)	138 (10)	<0.001*
**Aortic plaque, n (%)**	110 (3)	0 (0)	3 (0)	107 (8)	<0.001*
**Systolic blood pressure (mm Hg)**	125 ± 27	120 ± 37	123 ± 22	130 ± 24	<0.001*
**Diastolic blood pressure (mm Hg)**	74 ± 14	73 ± 13	73 ± 14	76 ± 15	<0.001*
**Heart rate (bpm)**	89 ± 26	85 ± 22	90 ± 27	90 ± 27	<0.001*
**Height (cm)**	162 ± 11	168 ± 8	162 ± 11	158 ± 10	<0.001*
**Weight (kg)**	64 ± 11	70 ± 13	63 ± 12	59 ± 12	<0.001*
**BMI**	24.4 ± 9.5	25.1 ± 12.9	24.3 ± 8.0	24.0 ± 8.0	0.016*
Echocardiographic data					
**LVEF (%)**	52±16	55±13	52±16	50±17	<0.001*
**LVEDD (mm)**	50±6	50±5	50±6	49±7	0.003*
**LVESD (mm)**	35±10	35±7	35±8	35±13	0.296

**LVEDV (mL)**	77±37	83±31	78±37	71±40	<0.001*
**LVESV(mL)**	42±31	42±26	78±37	71±40	0.155
**BSA (m^2)**	1.7±0.2	1.8±0.2	1.7±0.2	1.6±0.2	<0.001*
**LVEDD/BSA (mm/m^2)**	30±5	28±4	30±4	31±5	<0.001*
**LVESD/BSA (mm/m^2)**	21±6	19±4	21±5	22±7	<0.001*
**LVEDV/BSA (mL/m^2)**	46±21	46±17	46±20	45±24	0.209
**LVESV/BSA (mL/m^2)**	25±18	24±14	26±18	26±20	0.015*
**LA diameter (mm)**	45±16	43±15	46±15	45±16	0.003*
**LVMI (g/m^2)**	97±31	92±25	98±34	101±31	<0.001*
**RWT**	0.37±0.12	0.36±0.07	0.37±0.14	0.37±0.12	0.093
**LAVI (mL/m^2)**	49±22	43±19	50±22	54±25	<0.001*
**E/E'**	13±6	11±5	13±6	15±7	<0.001*
**Septal E' velocity (cm/s)**	7±3	8±4	7±2	6±2	<0.001*
**TR velocity max (m/s)**	2.5±0.4	2.4±0.4	2.5±0.4	2.6±0.5	<0.001*
**mDT (msec)**	118±114	117±64	119±104	118±139	0.974
**LVH, n(%)**	1,373 (33)	167 (16)	589 (33)	617 (44)	<0.001*

CHF, congestive heart failure; ICD, the International Statistical Classification of Diseases and Related Health Problem; TIA, Transient ischemic attack; BMI, body mass index; LVEF, ejection fraction; LVEDD, left ventricular end diastolic dimension; LVESD, left ventricular end systolic dimension; LVEDV, left ventricular end diastolic volume; LVESV, left ventricular end systolic volume; BSA, body surface area; LA, left atrial; LVMI, left ventricular mass index; LAVI, left atrial volume index; E/E', the ratio of early diastolic mitral inflow velocity to early diastolic velocity of the mitral annulus; RWT, relative wall thickness; mDT, mitral deceleration time; LVH, echocardiographic left ventricular hypertrophy

* $p < 0.05$

Supplementary Table S2. Univariate Cox regression models for CATE.

	CATE		
	HR	95% CI	p
**RFCA or CV**	0.15	0.021-1.07	0.059
**Anticoagulation**	0.30	0.20-0.45	<0.001
**Anticoagulation duration (months)**	0.94	0.91-0.96	<0.001
**Antiplatelet**	0.25	0.13-0.957	0.020
**ACEi/ARB**	0.28	0.18-0.43	<0.001
**BB**	0.41	0.27-0.63	<0.001
**CCB**	0.40	0.18-0.91	0.028
**Antiarrhythmic drugs**	0.09	0.02-0.35	<0.001
**Diuretics**	0.39	0.26-0.58	<0.001
**Electrocardiographic LVH**	1.12	0.88-1.64	0.257
**Height (cm)**	0.99	0.98-1.80	0.233
**Weight (kg)**	0.98	0.96-1.59	0.234
**ICD code or LVEF≤40%**	0.98	0.73-1.33	0.901
**LVEF≤40%**	1.12	0.79-1.61	0.521
**HFpEF**	1.04	0.64-1.67	0.881
**E/E' ≥ 11**	1.30	0.91-1.84	0.146
**LAVI > 34 ml/m^2**	1.24	0.85-1.82	0.261
**TR max PG ≥ 2.8 m/s**	1.28	0.85-2.04	0.218
**LVEF ≤ 40% and mDT ≥160 msec**	0.85	0.54-1.34	0.482
**DD**	1.19	0.73-1.94	0.486
**ICD code**	0.87	0.63-1.20	0.399

CATE, composite adverse thromboembolic events; LVH, left ventricular hypertrophy; RFCA: radiofrequency catheter ablation; CV, cardioversion; ACEi/ARB, angiotensin converting enzyme inhibitor/angiotensin receptor blocker; BB: beta blocker, CCB, calcium channel blocker

All other abbreviations are listed in Tables 1, 2 and 3.

Supplementary Table S3. Multivariable-adjusted Cox regression analysis of each CHA_2DS_2-VASc scores using different definitions for congestive heart failure in predicting composite thromboembolic events after excluding patients administered with anticoagulation

Definition of C criterion of CHA_2DS_2-VASc score	CATE (N = 143 events out of total N = 2752)		
	Adjusted HR	95% CI	P
**ICD code or LVEF≤40%**	1.12	0.90-1.39	0.330
**LVEF≤40%**	1.21	0.97-1.50	0.095
**HFpEF**	1.20	0.97-1.49	0.098
**E/E'≥11**	1.29	1.02-1.63	0.033
**LVEF≤40% and mDT≥160 msec**	1.24	0.90-1.72	0.192
**DD**	1.20	0.97-1.49	0.098
**ICD code**	1.06	0.85-1.32	0.601

Adjusted for cardioversion or RFCA, ACEi/ARBs, beta blocker, calcium channel blocker, antiarrhythmics, and diuretics

All other abbreviations are listed in Table 3.

Adjusted hazard ratio and 95% confidence intervals of other adjusted variables are shown in Supplementary Table S2.

*p<0.05

Supplementary Table S4. C-statistics of different definitions used for congestive heart failure of the CHA_2DS_2-VASc scores in predicting composite adverse thromboembolic events

C-statistics for CATE	C-statistic (95% CI)	P
CHA_2DS_2-VASc (E/E′≥11)	0.53 (0.48-0.59)	0.313
CHA2DS_2-VASc (LVEF≤40% and ICD code)	0.49 (0.43-0.55)	0.776
CHA_2DS_2-VASc (LVEF≤40%)	0.51 (0.45-0.56)	0.829
CHA_2DS_2-VASc (HFpEF)	0.50 (0.45-0.56)	0.897
CHA_2DS_2-VASc (ICD code)	0.51 (0.46-0.55)	0.755

All abbreviations are listed in Supplementary Tables S2 and S3.
